# Thoracoscopic Surgery in a Patient with Multiple Esophageal Carcinomas after Surgery for Esophageal Achalasia

**DOI:** 10.1155/2017/3272014

**Published:** 2017-08-30

**Authors:** Yuki Yamasaki, Tomoya Tsukada, Tatsuya Aoki, Yusuke Haba, Katsuhisa Hirano, Toshifumi Watanabe, Masahide Kaji, Koichi Shimizu

**Affiliations:** Department of Surgery, Toyama Prefectural Central Hospital, 2-2-78 Nishinagae, Toyama, Toyama 930-0975, Japan

## Abstract

We present a case in which we used a thoracoscopic approach for resection of multiple esophageal carcinomas diagnosed 33 years after surgery for esophageal achalasia. A 68-year-old Japanese man had been diagnosed with esophageal achalasia and underwent surgical treatment 33 years earlier. He was examined at our hospital for annual routine checkup in which upper gastrointestinal endoscopy showed a “0-IIb+IIa” lesion in the middle esophagus. Iodine staining revealed multiple irregularly shaped iodine-unstained areas, the diagnosis of which was esophageal carcinoma. Thoracoscopic subtotal esophagectomy was performed. Esophageal carcinoma may occur many years after surgery for esophageal achalasia, even if the passage symptoms have improved. So, long-term periodic follow-up is necessary for detection of carcinoma at an earlier stage.

## 1. Introduction

Esophageal achalasia (EA) is an idiopathic primary esophageal motor disorder characterized by impaired relaxation of the lower esophageal sphincter (LES) and loss of esophageal peristalsis [1]. The classic symptom of EA is dysphagia associated with regurgitation of undigested food. Some patients also experience weight loss, coughing, and chest pain. Although there is no definitive cure, current treatments aim to reduce LES pressure in order to relieve symptoms, improve esophageal emptying, and prevent further esophageal dilation [2]. Treatments include balloon dilation and Heller myotomy with Dor fundoplication.

Patients with EA are at an increased risk for developing squamous cell carcinoma (SCC) of the esophagus, which is thought to be caused by continuous exposure to stagnant ingested food [3]. It has been reported that the frequency of esophageal carcinoma (EC) decreases after surgery for EA [4], but there are several reports of patients diagnosed with EC even after undergoing surgery for EA [5–7]. To the best of our knowledge, this is the first case report of EC treated with thoracoscopic surgery after Heller-Dor procedure.

## 2. Case Presentation

**Figure 1:**
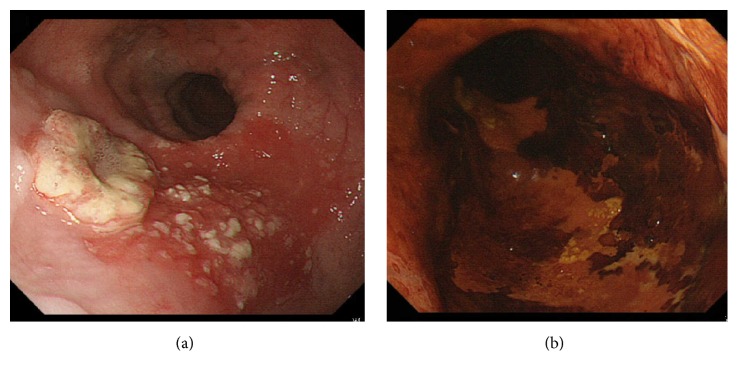
Upper gastrointestinal endoscopy showed a “0-IIb+IIa” lesion in the middle esophagus (a). Iodine staining revealed multiple irregularly shaped iodine-unstained areas (b).

**Figure 2:**
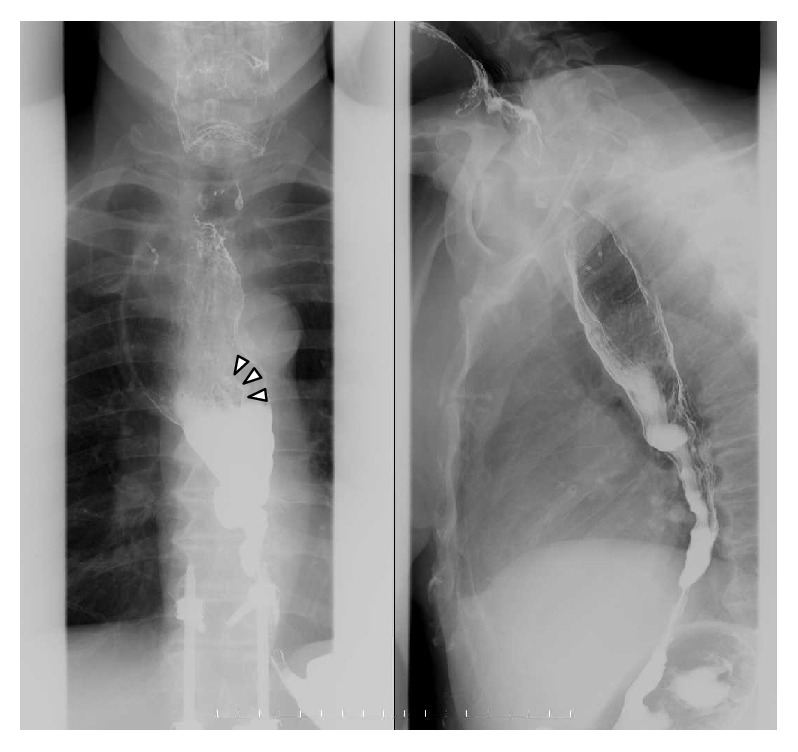
Barium esophagram showed esophageal dilation with a gradual tapering down to the gastroesophageal junction. There was a superficial protruding lesion in the midthoracic esophagus (arrowhead).

A 68-year-old Japanese man diagnosed with EA underwent surgical treatment 33 years ago. During an annual checkup, endoscopy revealed a “0-IIb+IIa” lesion in the middle esophagus (Figure 1(a)). The patient was referred to our hospital for further evaluation. Iodine staining revealed multiple irregularly shaped iodine-unstained areas spreading to the cervical esophagus (Figure 1(b)). These areas were biopsied and diagnosed as EC with invasion of the submucosal layer. Barium esophagram (Figure 2) showed esophageal dilation with a gradual tapering down to the gastroesophageal junction. Emptying of barium to the stomach was normal, owing to the previous surgery. There was a superficial protruding lesion in the midthoracic esophagus (arrowhead). A chest contrast-enhanced computerized tomography scan and positron emission tomography scan showed no lymph node or distant metastases. Early EC (clinical stage I; T1b(SM2)N0M0) was diagnosed based on the 11th edition of the Japanese Classification of Esophageal Carcinoma [6].

**Figure 3:**
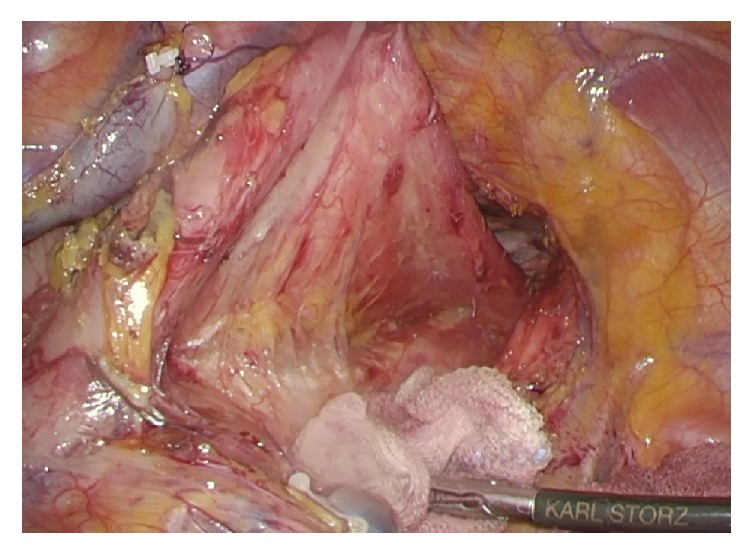
Intraoperative findings showed dilated esophagus. We secured the surgical field by elevating the esophagus dorsally and pushing down the trachea ventrally.

Thoracoscopic esophagectomy and open gastric pull-up reconstruction were planned. The thoracic procedure was performed with the patient in the prone position. Mediastinal lymph node dissection was performed, and the esophagus was resected to a level lower than the lesion. Dilation of the esophagus resulted in difficulty with securing the surgical field, but we were able to manage by changing the grasping parts frequently (Figure 3). The patient was then placed in the supine position and laparotomy was performed. Adhesions were encountered between the lateral segment of the liver and the lesser curvature of the stomach, likely a consequence of the previous abdominal surgery. Through a cervical neck incision, the proximal gastric pull-up was retrieved in the posterior mediastinal pathway and a cervical esophagogastric anastomosis was performed. Additionally, an enteral feeding tube was placed. The total surgical time was 464 minutes and the total estimated blood loss was 100 ml.

**Figure 4:**
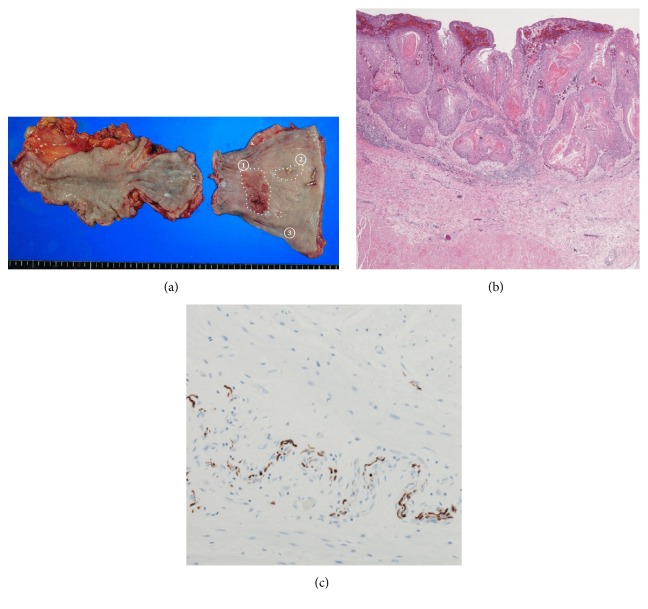
Resected specimen (a). There are three lesions (①–③) in the markedly dilated esophagus. Moderately differentiated SCC cells invaded the submucosal layer at elevated lesion (b). Loss of ganglion cells in the myenteric plexus throughout the length of the resected esophagus (c).

Histopathology showed a moderately differentiated SCC invading the submucosal layer (Figures 4(a)① and 4(b)), as well as two SCC invading the mucosal layer (Figure 4(a)②, ③). There were no lymph node metastases. The final stage was T1b(SM2)N0M0, stage IA. Other histopathologic findings included marked dilation of the upper-middle esophagus and loss of ganglion cells in the myenteric plexus throughout the length of the resected esophagus (Figure 4(c)) (Grade III at both the dilated part and the nondilated part, according to descriptive rules for achalasia of the esophagus [1]).

The patient's postoperative course was uneventful with the exception of mild left recurrent laryngeal nerve paralysis (Clavien-Dindo Grade I). He was discharged on postoperative day 20. Ten months after the surgery, the patient was well without evidence of disease recurrence.

All diagnostic procedures and therapy concerning the patient were carried out after informed consent had been obtained.

## 3. Discussion

EA is a primary disorder of esophageal motility and is regarded as a risk factor for SCC [3]. In a prospective study, Leeuwenburgh et al. reported that carcinoma develops on average 24 years (range: 10–43) after symptom onset [6]. However, there have been few reports on the results of follow-up after curative surgery for EA and it is unclear whether EC is frequent when EA has been treated successfully. Ellis Jr. et al. reported that the incidence of EC decreased to 0.3% in patients undergoing a second surgery for EA [4]. However, other reports have found that the incidence of carcinoma remained high despite surgery. For example, Arima et al. and Leeuwenburgh et al. reported that, respectively, 15.1 years and 11 years elapsed until the occurrence of EC after surgery for EA [7, 8]. Furthermore, Ota et al. reported the results of follow-up after curative surgery for EA. Thirty-two patients underwent long-term and periodic endoscopic follow-up. Esophageal SCC was detected in six patients (18%) and the average duration of follow-up until EC was seen after surgery for EA was 14.3 (5–40) years [5]. Carter and Brewer III reported that EC occurred early (15 months) after surgery for EA, but they considered that it was due to insufficient muscle layer incision [9]. These reports indicate that EC can occur many years after appropriate surgery for EA. In these patients including our case, the Heller-Dor operation was performed as surgical treatment for EA and no patient complained of passage symptoms. This suggests that the potential for malignant transformation persists even after surgery improves passage symptoms. Ribeiro Jr. et al. reported that the esophageal mucosa itself in EA appeared to be associated with malignant potential [10]. Also in our case, passage symptoms are improved after surgery for EA, but barium esophagram showed that esophageal spasm was diffusely observed in the middle and lower esophagus and the dilation of the upper esophagus still remained. From these findings of the barium esophagram, this case is presumed to be Chicago classification type III EA. In addition, ganglion cells were not observed in the myenteric plexus throughout the length of the resected esophagus, which suggests that this case is type III achalasia. Pandolfine et al., who proposed the Chicago classification by high resolution manometry (HRM), reported that type III EA is the most resistant to treatment [11]. Considering these facts, we cannot deny the possibility that chronic inflammation caused by asymptomatic stagnation of food and saliva has remained. HRM was not conducted in our case, but even in EA after surgery, HRM may be useful for monitoring the effect of the surgical treatment and as a predictor of EC. From this point of view, the possibility that food and liquid, including saliva, were retained in the esophagus with no symptoms and that EC occurred on the background of chronic inflammation cannot be denied.

Even after surgery for EA, in many cases, we consider that superficial EC (especially T1a-epithelium or T1a-lamina propria mucosae) can be treated with endoscopic submucosal dissection (ESD). There are several reports on ESD for early EC after surgery for EA [7, 12]. Our case was not considered for ESD, because there were multiple lesions spread across a wide area, and part of the lesion invaded deeply. There had been no report on thoracoscopic esophagectomy for EC after surgery for EA, but we could perform it in the prone position and open gastric pull-up reconstruction safely in the present case.

It is important to discover malignancy at earlier stage due to recognition of the risk of developing EC in the patients after surgery for EA and long-term periodic follow-up.

## References

[1] The Japan Esophageal Society (2012). *Descriptive Rules for Achalasia of the Esophagus*.

[2] Krill J. T., Naik R. D., Vaezi M. F. (2016). Clinical management of achalasia: current state of the art. *Clinical and Experimental Gastroenterology*.

[3] Brücher B. L. D. M., Stein H. J., Bartels H., Feussner H., Siewert J. R. (2001). Achalasia and esophageal cancer: incidence, prevalence, and prognosis. *World Journal of Surgery*.

[4] Ellis Jr. F. H., Crozier R. E., Gibb S. P. (1986). Reoperative achalasia surgery. *The Journal of Thoracic and Cardiovascular Surgery*.

[5] Arima M., Kouzu T., Arima H. (1995). Superficial esophageal cancer (m1) associated with postoperative achalasia of the esophagus, report of a case. *Stomach and Intestine*.

[6] Leeuwenburgh I., Scholten P., Alderliesten J. (2010). Long-term esophageal cancer risk in patients with primary achalasia: A prospective study. *American Journal of Gastroenterology*.

[7] Ota M., Narumiya K., Kudo K. (2016). Incidence of esophageal carcinomas after surgery for achalasia: usefulness of long-term and periodic follow-up. *American Journal of Case Reports*.

[8] The Japan Esophageal Society (2015). *Japanese Classification of Esophageal Cancer*.

[9] Carter R., Brewer L. A. (1975). Achalasia and esophageal carcinoma. Studies in early diagnosis for improved surgical management. *The American Journal of Surgery*.

[10] Ribeiro U., Posner M. C., Safatle-Ribeiro A. V., Reynolds J. C. (1996). Risk factors for squamous cell carcinoma of the oesophagus. *British Journal of Surgery*.

[11] Pandolfino J. E., Kwiatek M. A., Nealis T., Bulsiewicz W., Post J., Kahrilas P. J. (2008). Achalasia: a new clinically relevant classification by high-resolution manometry. *Gastroenterology*.

[12] Chino O., Shimada H., Kise Y. (2008). Early carcinoma of the esophagus associated with achalasia treated by endoscopic mucosal resection. *The Tokai Journal of Experimental and Clinical Medicine*.

